# The D-Lactate Dehydrogenase from *Sporolactobacillus inulinus* Also Possessing Reversible Deamination Activity

**DOI:** 10.1371/journal.pone.0139066

**Published:** 2015-09-23

**Authors:** Lingfeng Zhu, Xiaoling Xu, Limin Wang, Hui Dong, Bo Yu

**Affiliations:** 1 CAS Key Laboratory of Microbial Physiological and Metabolic Engineering, Institute of Microbiology, Chinese Academy of Sciences, Beijing, 100101, PR China; 2 Key Laboratory of Tianjin Radiation and Molecular Nuclear Medicine, Institute of Radiation Medicine, Chinese Academy of Medical Sciences & Peking Union Medical College, Tianjin, 300192, PR China; 3 Institute of Ageing Research, School of Medicine, Hangzhou Normal University, Hangzhou, 311121, PR China; 4 University of Chinese Academy of Sciences, Beijing, 100049, PR China; Wageningen University, NETHERLANDS

## Abstract

Hydroxyacid dehydrogenases are responsible for the conversion of 2-keto acids to 2-hydroxyacids and have a wide range of biotechnological applications. In this study, a D-lactate dehydrogenase (D-LDH) from a *Sporolactobacillus inulinus* strain was experimentally verified to have both the D-LDH and glutamate dehydrogenase (GDH) activities (reversible deamination). The catalytic mechanism was demonstrated by identification of key residues from the crystal structure analysis and site-directed mutagenesis. The Arg^234^ and Gly^79^ residues of this enzyme play a significant role in both D-LDH and GDH activities. His^295^ and Phe^298^ in DLDH744 were identified to be key residues for lactate dehydrogenase (LDH) activity only whereas Tyr^101^ is a unique residue that is critical for GDH activity. Characterization of the biochemical properties contributes to understanding of the catalytic mechanism of this novel D-lactate dehydrogenase enzyme.

## Introduction

Hydroxyacid dehydrogenases are responsible for stereospecific conversion of 2-keto acids to 2-hydroxyacids [1, 2]. Given the wide range of biotechnological applications in industry [3, 4, 5, 6], it is of significant biological interest to gain in-depth insights into the enzymes. Lactate dehydrogenase (LDH), one of the hydroxyacid dehydrogenases, is a terminal glycolytic enzyme. It catalyzes the (reversible) reduction of pyruvate to lactic acid, concomitant with the oxidation of NADH to NAD^+^ [2]. A hydride ion is transferred from NADH to pyruvate during this reaction; reversely, it is transferred to NAD^+^ from lactate. A unifying feature of all previously reported lactate dehydrogenases is their strict specificity for NAD^+^ as a cofactor [7].

Glutamate dehydrogenases (GDHs) are found in nearly every organism and play a fundamental role in nitrogen and carbon metabolism [8]. In the oxidative deamination reaction, GDH links amino acid metabolism to the tricarboxylic acid cycle by converting L-glutamate to 2-oxoglutarate (α-ketoglutarate), whereas the reductive amination reaction supplies nitrogen for several biosynthetic pathways [9]. GDH belongs to the amino acid dehydrogenase superfamily, members of which display differential substrate specificity as well as valine, leucine and phenylalanine dehydrogenases [8]. Bacterial glutamate dehydrogenases vary in their coenzyme preference: NAD^+^ specificity, NADP^+^ specificity, and a few which have dual cofactor specificity [10].

There are rare reports about the bifunctional activities of a lactate dehydrogenase. Vancomycin resistance proteins H (VanH) in the D-isomer-specific 2-hydroxyacid dehydrogenase family is the only reported bifunctional enzyme that has additionally D-lactate dehydrogenase activity in nature [11]. We previously reported that a novel D-lactate dehydrogenase from *Sporolactobacillus inulinus* CASD (DLDH744) could use both NADH and NADPH and even with a preference for NADPH as the coenzyme, which is different from the coenzyme utilization of all previously reported LDHs [12]. Here, the DLDH744 was also experimentally proven to possess both D-lactate and glutamate dehydrogenase activities. The structure-guided mutagenesis study was applied to demonstrate the catalytic mechanism of this novel enzyme.

## Materials and Methods

### Bacterial strains and protein expression

*S*. *inulinus* CASD was used as the source for cloning DLDH744 gene [13]. *Escherichia coli* strains were grown in Luria-Bertani (LB) medium aerobically and set on a rotary shaker. *E*. *coli* DH5α (Tiangen, Beijing, China) was used for general cloning, while *E*. *coli* BL21 (DE3) (Tiangen, Beijing, China) was used for protein expression. Plasmids pMD 19-T (TaKaRa, Dalian, China) and pET-28a(+) (Novagen) were used as vectors. The gene expression, protein purification, X-ray crystallography and structure determination were as previously described [12].

### Enzymatic activity assay procedures

After purification, the activity of glutamate dehydrogenase was assayed photometrically (absorbance at 340 nm) following NAD(P)H production according to the formula, glutamate+ NAD(P)^+^ + H_2_O ↔ á-ketoglutarate + NAD(P)H + NH_4_^+^. The assay mixture (400 μL) contained 100 mM PBS (pH 5.5), 10 mM glutamate, 0.2 mM NAD(P)^+^, and 20 μL of enzyme [14]. The reverse reaction assay mixture (400 μL) contained 100 mM PBS (pH 5.5), 10 mM á-ketoglutarate, 10 mM NH_4_Cl, 0.2 mM NAD(P)H, and 20 μL of enzyme. The protein concentration of the enzyme stock solution was 3 mg/mL. One unit was defined as the amount of enzyme converting 1 μmol of NAD(P)H per min. Specific activity has been expressed as units per mg protein.

### Kinetic studies of DLDH744

The kinetic constants of purified enzyme were calculated using 10 μg DLDH744 enzyme in 100 mM PBS (pH 5.5) at 30°C. NADH or NADPH was used as cofactor with the concentration of 0.2 mM. The concentrations of pyruvate, phenylpyruvate and glutamate used were measured from 0.5 mM to 20 mM, respectively. The ΔOD_340_ between 1 min and 2 min were chosen as the initial rate for kinetic analysis. The resulting kinetic parameters were calculated from multiple measurements and extracted by using ChemSW^TM^ software.

### Site-directed mutagenesis

The mutant strains used in this study were constructed by site-directed PCR mutagenesis using *Pyrobest* DNA polymerase (TaKaRa, Dalian, China) [15]. The primers used for the introduction of the mutations are shown in Table 1. After the PCR reaction, the remaining template was digested with the restriction enzyme DpnI (TaKaRa, Dalian, China) at 37°C for 2 h. The DpnI-digested PCR product was then purified and transformed to *E*. *coli* BL21(DE3) for gene expression. The cells were spread on LB agar plates containing 30 μg/mL kanamycin. Single clones were selected for identification using gene sequencing.

**Table 1:** Primers used for site-directed mutagenesis of DLDH744.

Primer	Sequence (5’−3’)
G79A+	CTCGCGTACAGCCGCGTACGATATGATC
G79A−	GATCATATCGTACGCGGCTGTACGCGAG
Y101A+	CAATGTGCCGGCTGCGTCGCCGAACTC
Y101A−	GAGTTCGGCGACGCAGCCGGCACATTG
R234A+	CATCAATGCATCGGCGGGCCCGGTCGTCGATAC
R234A−	GTATCGACGACCGGGCCCGCCGATGCATTGATG
E263A+	GATACACTGAACGGTGCGGAGCACTTCTTC
E263A−	GAAGAAGTGCTCCGCACCGTTCAGTGTATC
H295A+	CTGATTACGCCGGCGATTGGTTTCTACAC
H295A−	GTGTAGAAACCAATCGCCGGCGTAATCAG
F298A+	GATTACGCCGCATATTGGTGCGTACACCAACAAAG
F298A−	CTTTGTTGGTGTACGCACCAATATGCGGCGTAATC
M307A+	CGTGCAAAATGCGGTTGAGATCAGCCTG
M307A−	CAGGCTGATCTCAACCGCATTTTGCACG

+, sense sequence;

−, anti-sense sequence.

The codons with underline indicated the mutation site.

### Phylogenetic analysis

The sequences of all the members belonging to the D-isomer-specific 2-hydroxyacid dehydrogenase family and the structurally similar NAD^+^-dependent formate dehydrogenase, phosphate dehydrogenase and L-alanine dehydrogenase were selected for the phylogenetic analysis. The resulting 40 sequences along with the sequence of DLDH744 were aligned using the multiple sequence alignment program ClustalX2 [16]. The phylogenetic analysis was conducted by using MEGA6 software with full-length amino acid sequences. The neighbor-joining method was used to draw phylogenetic tree.

### Analytical methods

Glutamic acid was measured by HPLC (Agilent 1260 series) equipped with an Agilent Eclipse XDB-C18 column (250 × 4.6 mm, 5 μm) and a variable-wavelength detector at 205 nm. The mobile phase consisted of 10 mM KH_2_PO_4_ (pH 2.5) and acetonitrile at a ratio of 95:5 (v/v) and a flow rate of 0.8 mL/min at 30°C.

## Results

### Glutamate dehydrogenase (GDH) activity of DLDH744

**Fig 1:**
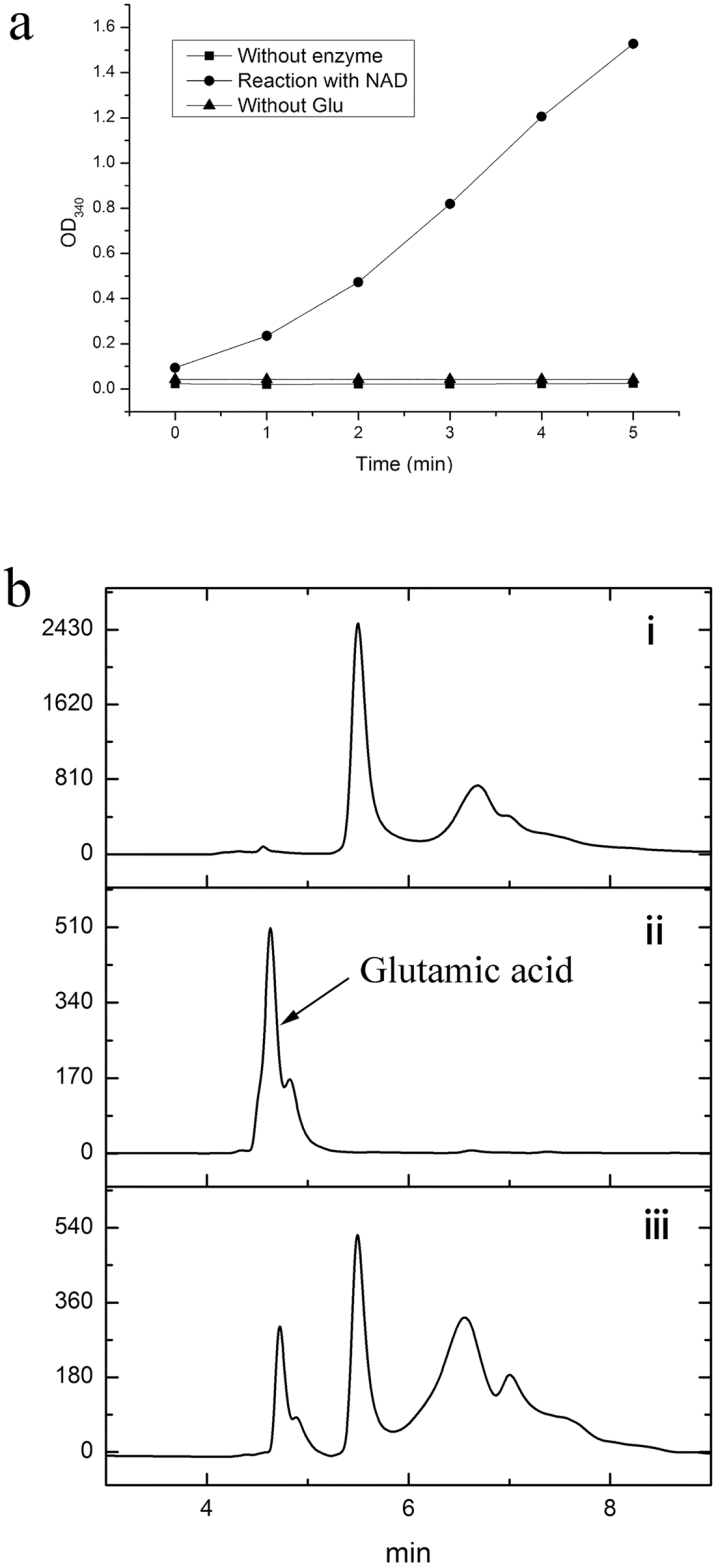
Analysis of the glutamate dehydrogenase activity of DLDH744. (a) Analysis of the enzymatic activity using a UV spectrophotometer. (Filled circle) Reaction with NAD^+^ as coenzyme. (Filled block) Reaction without DLDH744. (Filled triangles) Reaction without glutamate substrate. (b) Analysis of the reaction product using HPLC. (i) Control. (ii) Glutamic acid standard. (iii) Reaction solution with substrates and enzyme DLDH744.

DLDH744 is a homodimeric protein with a length of 335 amino acids. The enzyme was confirmed to be a typical D-lactate dehydrogenase based on the activity detected with substrate pyruvate and coenzyme NAD(P)H [12]. The apparent *K*_m_ value for pyruvate was determined to be 3.4 ± 0.02 mM with a *k*_cat_ value of 512.2 ± 7.6 /min in this study. Upon submission of the draft genome sequence of *S*. *inulinus* strain CASD to NCBI database, the gene fragment of DLDH744 was predicted to be a bifunctional enzyme with the possible amino acid dehydrogenase activities. Therefore, we tested DLDH744 activity for various amino acids, including glutamate, aspartate, proline, valine, leucine, isoleucine, alanine, and phenylalanine. Only glutamate oxidation activity was found while the other amino acid oxidation activities were not detected. DLDH744 showed strong glutamate oxidation activity (Fig 1a). The apparent *K*_m_ value for glutamate oxidation was 5.7 ± 0.09 mM, which is higher than that of pyruvate. The *k*_cat_ value for glutamate oxidation was calculated to be 22.3 ± 0.1 /min. The reverse reaction product glutamic acid was also confirmed by HPLC when using NADH as a coenzyme and á-ketoglutarate with NH_4_^+^ as substrates (Fig 1b). No product of hyroxyglutarate was detected in the absence of ammonia (data not shown). Additionally, phenylpyruvate was also experimentally confirmed to be a substrate for DLDH744 with an apparent *K*_m_ value of 3.32 ± 0.08 mM for phenylpyruvate, which is very close to that of pyruvate. Only D-phenyllactic acid was detected as the product from phenylpyruvate, indicating DLDH744 is a strict D-2-hydroxyacid dehydrogenase. Both NAD^+^ and NADP^+^ could serve as coenzymes for GDH activity (data not shown), which is consistent with previously reported LDH activity [12]. The specific activity of DLDH744 was 7.5 ± 0.17 U/mg for the LDH activity and 0.89 ± 0.01 U/mg for the GDH activity.

### The residues responsible for stabilizing the dimer interface

**Fig 2:**
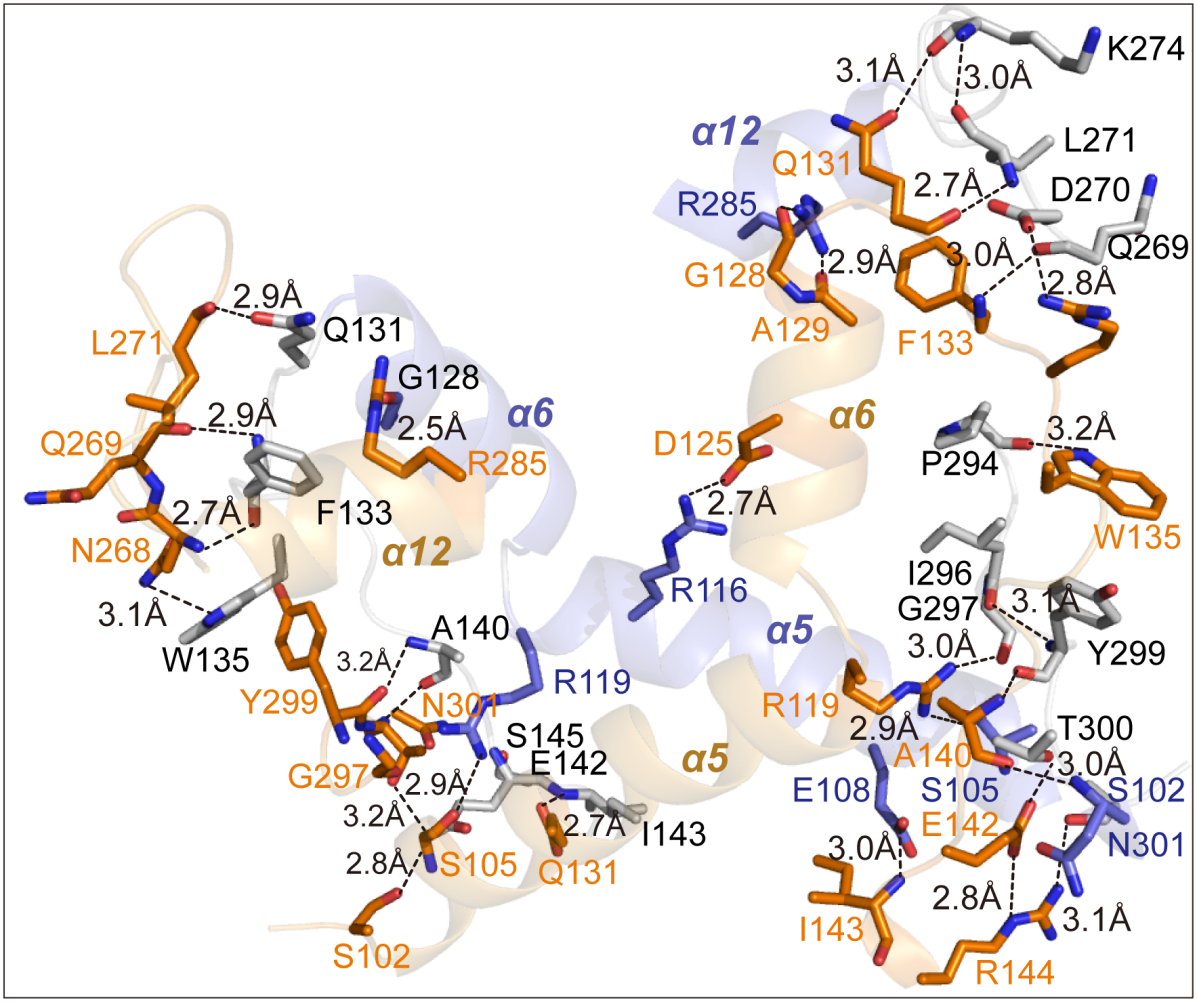
The residues responsible for stabilizing the dimer interface. The subunits of the homo-dimer are shown in blue and orange respectively. The secondary structures located at the dimer interface are shown in ribbon and the key amino acid residues that mediate the dimer formation are shown as dashed lines.

To further investigate this novel enzyme, the complete tertiary structure of DLDH744 in complex with NAD^+^ was determined (PDB accession code: 4XKJ). The structure of DLDH744 was determined by molecular replacement method and refined at 3.15 Å-resolution as previously described [12]. Two identical monomers of DLDH744 were (with Cα atoms r.m.s. difference of 1.339 Å) associated at the cofactor binding domain to form the homo-dimer in one asymmetric unit. The dimer interface is stabilized mainly by hydrogen-bonding interactions between residues from α helices α5(Ser^102^-Arg^119^), α6(Leu^121^-Glu^130^), α12(Glu^279^-Arg^285^) and connecting loops (Fig 2). Overall, the α5 helices from two subunits are interacting with each other, and α6 and α12 are associated by hydrogen bonds to bring the two subunits together to form the homo-dimer. In the dimer interface, the amino group of Arg^116^ at α5 forms a hydrogen bond with the hydroxyl group of Asp^125^ at α5 of another subunit (2.7 Å), the amino group of Arg^119^ form a hydrogen bond with Gly^297^ (2.8 Å) and Ser^105^ (2.9 Å), and the main chain oxygen of Gly^128^ forms interacts with the amino group of Arg^285^ in α12 (2.5 Å), carboxyl group of Gln^131^ is hydrogen bonding with the main chain oxygen of Leu^271^ (2.9 Å). The main chain oxygen and nitrogen atoms of Phe^133^ form a hydrogen bond with Gln^269^ (2.9 Å), Asn^268^ (2.7 Å) in the loop region of another subunit. Side chain of Trp^135^ interacts with Asn^268^ (3.1Å). Ala^140^ forms a hydrogen bond with main chain of Tyr^299^ (3.2 Å) and Asn^301^ (2.7 Å), while the side chain of Asn^301^ is further stabilized by a hydrogen bond with Ser^145^ (3.1 Å). The hydrogen bonds between the carboxyl group of Glu^142^ and hydroxyl group of Ser^102^ (2.8 Å), and the main chain nitrogen atom of Ile^143^ and hydroxyl group of Glu^108^ (3.0 Å) stabilize the conformation of the α5.

### Identification of DLDH744 active sites by structural analysis

**Fig 3:**
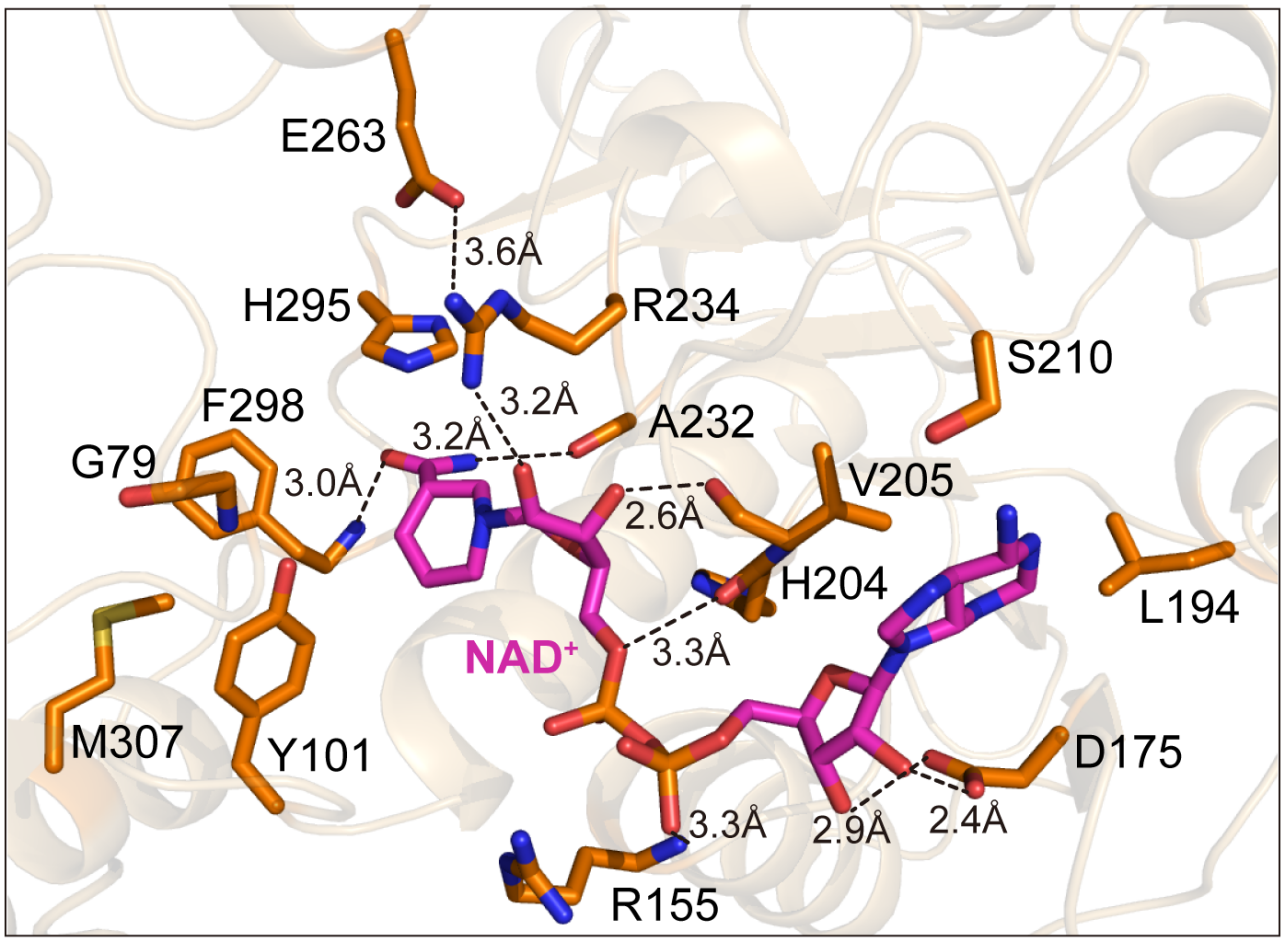
The coenzyme NAD^+^ binding sites of the DLDH744 in the active center. The key amino acids necessary for NAD^+^ binding are shown in sticks in orange. The hydrogen bonding interactions between these amino acid residues and NAD^+^ are shown in dashed lines, with the distance labeled.

As DLDH744 was demonstrated to be a novel bifunctional enzyme, it is interesting to explore the catalytic mechanism from the structure analysis. Although it has both LDH and GDH activities, the protein structure shows that only one active center was found in DLDH744. The cofactor NAD^+^ molecule binds in the pocket located on top of the cofactor binding domain. The NAD^+^ molecule adopts an extended conformation where the nicotinamide and ribose rings are approximately perpendicular to the adenine ring. The dihydronicotinamide group is extended into a pocket formed by His^295^, Arg^234^, Gly^297^, Phe^298^ and Tyr^101^ (Fig 3). NADH could be converted to NAD^+^ when the nicotinamide ring donates a proton. Meanwhile, pyruvate or á-ketoglutarate gets the proton and forms lactic acid or glutamic acid in the same place. The residues around the nicotinamide ring of NADH in the active center of the crystal structure should be essential for enzyme reaction.

By comparing with other NAD-dependent lactate dehydrogenases [17], His^295^ is supposed to function as the internal acid-base catalyst in dehydrogenase activity; Glu^263^ stabilizes the protonated form of His^295^, and Arg^234^ forms a hydrogen bond with the nicotinamide ribose ring (3.2 Å). Additionally, the main chain nitrogen atom of Phe^298^ forms a hydrogen bond with the carbonyl group of nicotinamide ring. Gly^79^ is located in the entrance of the substrate entry. But Tyr^101^ and Met^307^ stay inside the substrate entry way, and may select the substrate at the cleft between the substrate binding domain and cofactor binding domain. Phe^298^, Tyr^299^ and Trp^135^ further form a hydrophobic triad near the active site, and possibly determine the substrate specificity, such as lactate or glutamate, by steric hindrance near the nicotinamide ring.

### Site mutation study revealing the key active site residues

**Fig 4:**
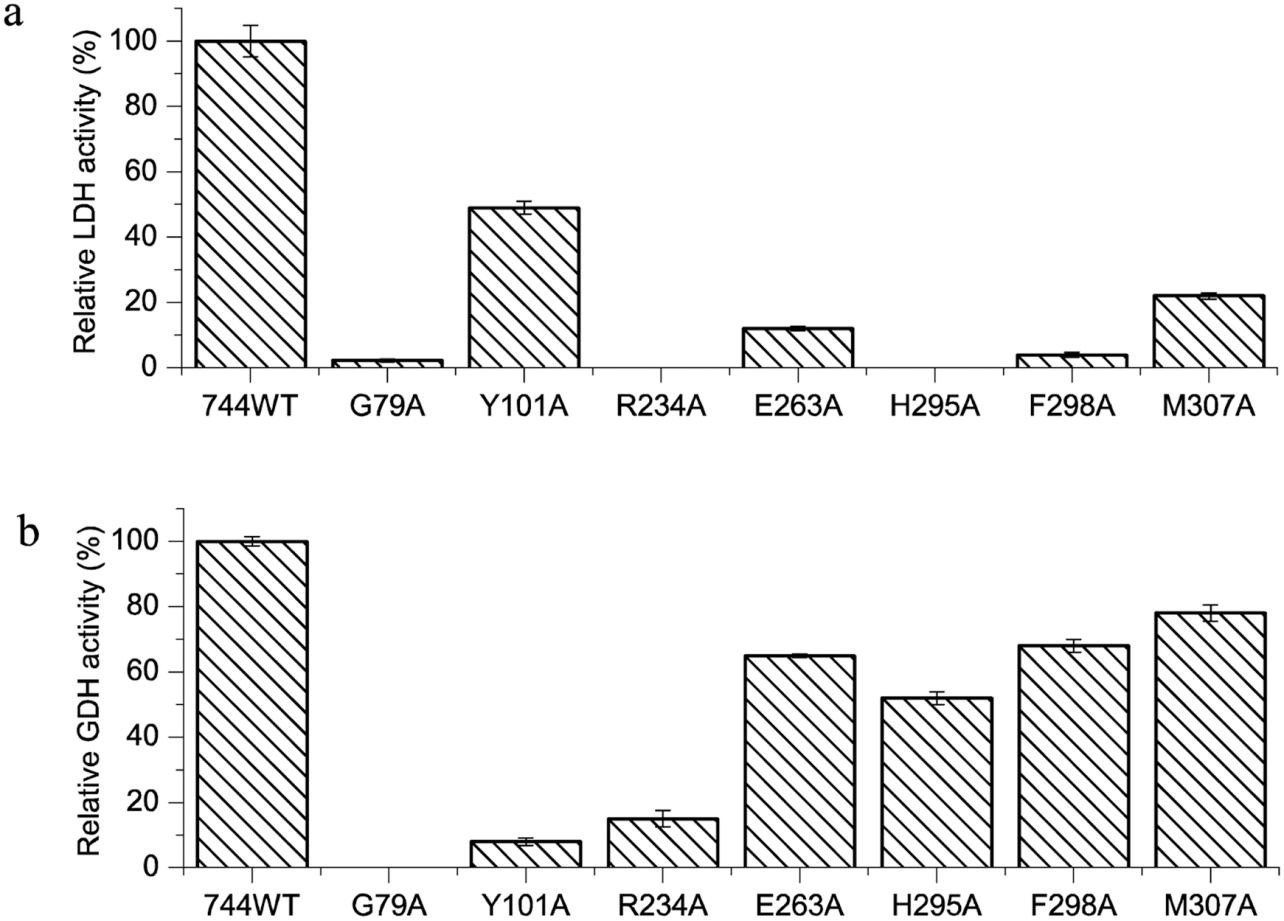
The active residue sites of DLDH744. (a) The activities of mutant lactate dehydrogenases. (b) The activities of glutamate dehydrogenase of the mutant strains. Data points and error bars represent the means and standard deviations of three parallel replicates, respectively.

Based on the crystal structure, above amino acids around the nicotinamide ring were chosen to study their roles in the LDH and/or GDH activities. Site-directed mutagenesis was conducted and the resulting enzymatic activities were evaluated. The mutations that showed dramatic influences on the LDH and/or GDH activities are shown in Fig 4. The LDH activities of Arg^234^, Gly^79^, Glu^263^, His^295^, Phe^298^ and Met^307^ mutants were dramatically reduced, indicating the significant role of these residues in D-LDH activity, which are consistent with the structural analysis. Additionally, the two residues Arg^234^ and Gly^79^ play a significant role in both LDH and GDH activities. Tyr^101^ is the one that was only critical for GDH activity.

### Phylogenetic relationship

**Fig 5:**
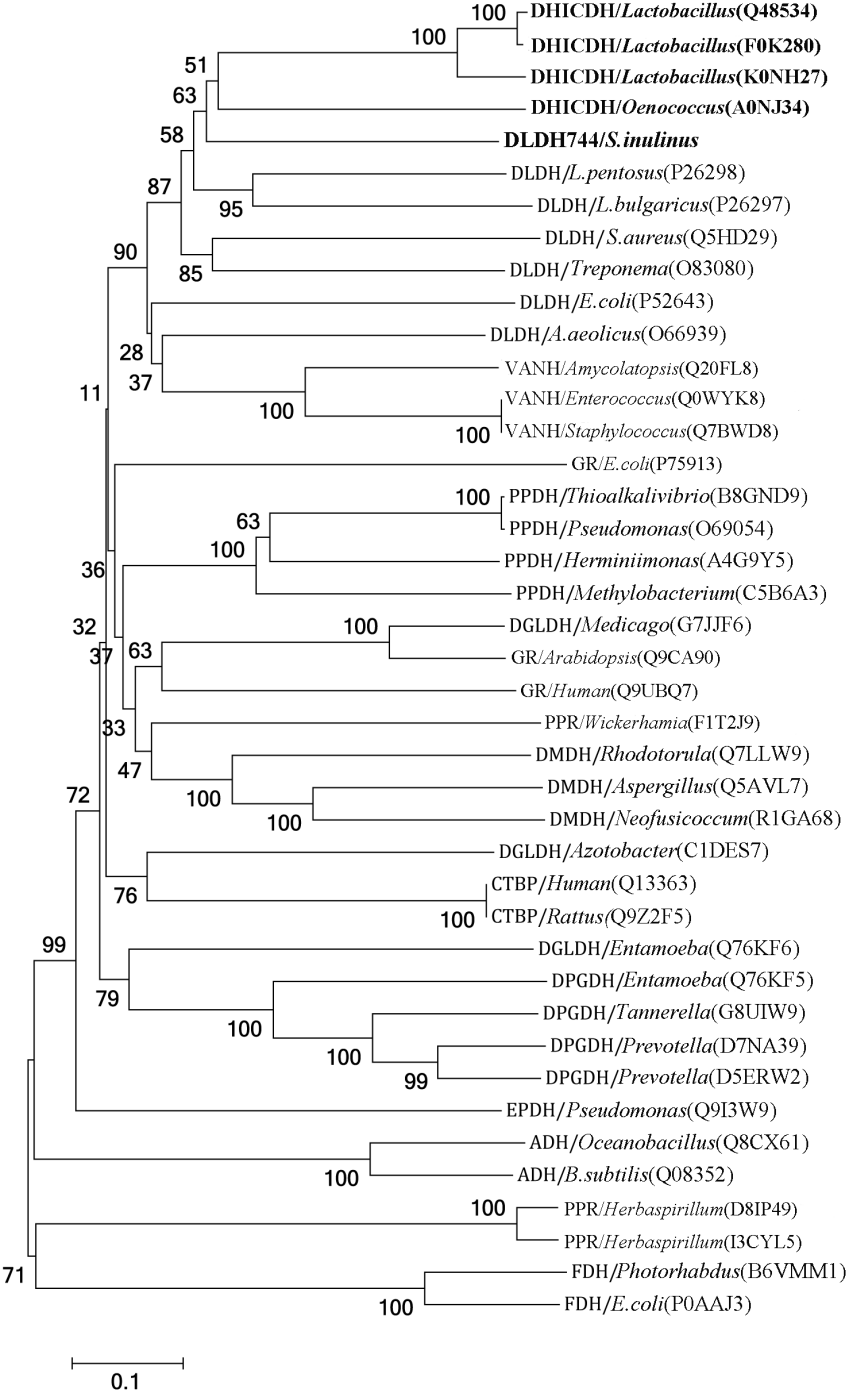
Phylogenetic analysis of DLDH744 with the other enzymes in D-isomer-specific 2-hydroxyacid dehydrogenase family and some non-D-specific 2-hydroxyacid dehydrogenases. The accession number of each enzyme in uniprot database (http://www.uniprot.org) is provided in brackets. Abbreviations: D-lactate dehydrogenase (DLDH), D-erythronate-4-phosphate dehydrogenase (DEPDH), D-hydroxyisocaproate dehydrogenase (DHICDH), D-phosphoglycerate dehydrogenase (DPGDH), D-glycerate dehydrogenase (DGLDH), D-mandelate dehydrogenase (DMDH), vancomycin-resistant protein H (VANH), phenylpyruvate reductase (PPR), glyoxylate reductase (GR), 2-ketopantoate reductase (KPR), transcriptional co-repressor CtBP (CTBP), formate dehydrogenase (FDH), phosphate dehydrogenase (PPDH) and L-alanine dehydrogenase (ADH).

There is no significant similarity between DLDH744 and any GDHs present in the UNIPROT database, making the referential study of DLDH744 from GDH unreliable. Then we just chose the enzymes from D-isomer-specific 2-hydroxyacid dehydrogenase family to investigate the evolutionary relationships. D-isomer-specific 2-hydroxyacid dehydrogenase family includes D-lactate dehydrogenase (DLDH), D-erythronate-4-phosphate dehydrogenase (DEPDH) [18], D-hydroxyisocaproate dehydrogenase (DHICDH) [19], D-phosphoglycerate dehydrogenase (DPGDH) [20], D-glycerate dehydrogenase (DGLDH) [21], D-mandelate dehydrogenase (DMDH) [22], vancomycin-resistant protein H (VANH) [23], phenylpyruvate reductase (PPR) [24], glyoxylate reductase (GR) [25], 2-ketopantoate reductase (KPR) [26] and transcriptional co-repressor CtBP (CTBP) [27]. Enzymes from this family share a common characteristic: the chiral product of the reaction is the D-form, and they generally exhibit strict specificity toward NAD^+^ except that GR and KPR are NADP^+^-dependent while PPR and VANH preferentially use NADPH. However, the core fold of all the above enzymes is structurally conserved [28]. The NAD^+^-dependent formate dehydrogenase (FDH) [29], phosphate dehydrogenase (PPDH) [30] and L-alanine dehydrogenase (ADH) [31] also share the structural similarities with the D-isomer-specific 2-hydroxyacid dehydrogenase family. Fig 5 shows a phylogenetic tree of DLDH744 and all the above enzymes. Interestingly, DLDH744 is a little different from the other D-lactate dehydrogenases, while it is more phylogenetically close to D-hydroxyisocaproate dehydrogenase. These results indicated that there is a diversity of substrate patterns in the D-isomer-specific 2-hydroxyacid dehydrogenase family, although they share the similar structure.

## Discussion

D-LDH and GDH belong to two distinct families: D-2-hydroxyacid dehydrogenases and Glu/Leu/Phe/Val dehydrogenases, respectively. As DLDH744 possesses both activities, the comparison of protein structures between DLDH744 and GDHs was first investigated. However, the similarities between DLDH744 and GDH were rather low on the amino acid sequence level. Consequentially, the study was shifted to investigate the protein structure to reveal the key sites to explore the mechanism.

Gly^79^, Arg^234^, His^295^ and Phe^298^ in DLDH744 were identified to be key residues for lactate dehydrogenase (LDH) activity. Notably, Glu^263^ and Met^307^ are also important residues for LDH activity since the relative activities of the mutated DLDH744 were around 5% ~ 20% of that of wild enzyme, respectively. In previously reported L-lactate dehydrogenases, His^195^ plays a crucial role in enzyme catalysis [32, 33]. It forms a hydrogen bond with the carbonyl group of pyruvate or the hydroxyl group of lactate, thus polarizing the group and making it prone to receiving or donating a hydride ion. This residue also acts as an acid/base group by donating (or abstracting) a proton to the pyruvate carbonyl group or the lactate hydroxyl group [34]. Therefore, it is important that this histidine residue is able to switch between protonated and deprotonated forms for the reversibility of the reaction. In DLDH744, the corresponding histidine residue is His^295^. This residue is highly conserved in the sequence alignment of D-2-hydroxy acid dehydrogenases. Structural studies on D-LDH and members of the D-2-hydroxyacid dehydrogenase family have confirmed that the corresponding histidine residue forms a hydrogen bond with the carbonyl group of pyruvate [35, 36] and is a proton donor for D-LDH [37]. Additionally, by comparing with other NAD-dependent dehydrogenases, Glu^263^ stabilizes the protonated form of His^295^ and increases its pKa [38]. The main chain nitrogen atom of Phe^298^ forms hydrogen bond with the carbonyl group of nicotinamide ring, then mutation of Phe^298^ to Ala decreased the NAD^+^ binding affinity. Furthermore, Phe^298^ and Met^307^ may be structurally responsible for discrimination of the substrates. Thus, mutation of His^295^, Glu^263^, Met^307^ and Phe^298^ to alanine residue all decreased the LDH activity.

In contrast to the LDH activity, only mutation of Gly^79^, Tyr^101^ and Arg^234^ decreased the GDH activity. Gly^79^ and Arg^234^ were reported to be involved in substrate binding and mediating the orientation of the substrate [37]. Gly^79^ was observed to be located at the substrate entry way while Arg^234^ is structurally found to function in stabilizing the nicotinamide ribose ring by hydrogen bonding interaction (3.2 Å) in this study. The LDH and GDH activities of the Arg^234^ and Gly^79^ mutants were both markedly decreased confirming the role of these residues in substrate binding. The substrate molecule is oriented face-on and directly adjacent to the nicotinamide ring of the coenzyme, where it is ideally positioned for transfer of a hydride ion during catalysis. Orientation of the substrate is very important for the enzyme reaction, thus Arg^234^ and Gly^79^ are essential for both LDH and GDH activities.

Lys^125^ of GDH have been found to act as acid/base groups by donating (or abstracting) a proton, similar to the function of His^295^ in LDHs [39]. However, the lysine residues of DLDH744 are located far from the active center and are therefore less likely to be responsible for GDH activity. Gunka et al. [40] reported that the tyrosine residue was crucial for activity of the glutamate dehydrogenase RocG from *Bacillus subtilis* since the activity of the Y158H mutant was severely reduced. Structural analysis showed that Tyr^101^ is located structurally on top of the nicotinamide ring and at the substrate entry way in DLDH744. It is plausible that this residue is available to make contact with substrates and act as a key residue for GDH activity. Consistently, the subsequent experiments verified Tyr^101^ of DLDH744 as a unique important residue for GDH activity. Interestingly, DLDH744 is different from other D-lactate dehydrogenases and it is much phylogenetically closer to D-hydroxyisocaproate dehydrogenase family. DLDH744 exhibit high activity towards 2-ketoacids with bigger substrate (α-ketoglutarate) or much larger aromatic side chain phenylpyruvate, indicating DLDH744 might also have a larger space in the active center which gives an explanation why DLDH744 could accept the larger substrates.

## Conclusions

The D-lactate dehydrogenase from a *Sporolactobacillus inulinus* strain was experimentally verified to have both lactate dehydrogenase and glutamate dehydrogenase activities. The biochemical properties of the enzyme were illustrated by X-ray crystal structural characterization and site-mutation analyses. Characterization of the active residues of this enzyme contributes to the mechanistic understanding of the activities.
